# EZH2-mediated epigenetic silencing of tumor-suppressive let-7c/miR-99a cluster by hepatitis B virus X antigen enhances hepatocellular carcinoma progression and metastasis

**DOI:** 10.1186/s12935-023-03002-9

**Published:** 2023-09-09

**Authors:** Chen-Shiou Wu, Yi-Chung Chien, Chia‐Jui Yen, Jia-Yan Wu, Li-Yuan Bai, Yung-Luen Yu

**Affiliations:** 1 Institute of Translational Medicine and New Drug Development, Taichung, 40402 Taiwan; 2 Center for Molecular Medicine, China Medical University Hospital, Taichung, 40402 Taiwan; 3 Graduate Institute of Biomedical Sciences, China Medical University, Taichung, 40402 Taiwan; 4 Division of Hematology and Oncology, Department of Internal Medicine, College of Medicine, National Cheng Kung University Hospital, National Cheng Kung University, Tainan, 70403 Taiwan; 5 Division of Hematology and Oncology, China Medical University Hospital, Taichung, 40402 Taiwan; 6 Department of Medical Laboratory Science and Biotechnology, Asia University, Taichung, 41354 Taiwan

**Keywords:** EZH2, let-7c, miR-99a, Hepatitis B Virus X Antigen, Hepatocellular Carcinoma

## Abstract

**Background:**

Hepatitis B virus (HBV)-encoded X antigen, HBx, assists in the development of hepatocellular carcinoma (HCC) through complex mechanisms. Our results provide new insights into the EZH2 epigenetic repression of let-7c that promotes HCC migration induced by HBx. Thus, let-7c and HMGA2 represent key diagnostic markers and potential therapeutic targets for the treatment of HBV-related HCC.

**Results:**

We investigated the epigenetic regulation of let-7c, an important representative miRNA in liver tumor metastasis, in human HCC cells to verify the effect of HBx. Based on quantitative PCR (qPCR) of mRNA isolated from tumor and adjacent non-tumor liver tissues of 24 patients with HBV-related HCC, EZH2 expression was significantly overexpressed in most HCC tissues (87.5%). We executed a miRNA microarray analysis in paired HBV-related HCC tumor and adjacent non-tumorous liver tissue from six of these patients and identified let-7c, miR-199a-3p, and miR-99a as being downregulated in the tumor tissue. Real-time PCR analysis verified significant downregulation of let-7c and miR-99a in both HepG2X and Hep3BX cells, which stably overexpress HBx, relative to parental cells. HBX enhanced EZH2 expression and attenuated let-7c expression to induce HMGA2 expression in the HCC cells. Knockdown of HMGA2 significantly downregulated the metastatic potential of HCC cells induced by HBx.

**Conclusions:**

The deregulation of let-7c expression by HBx may indicate a potential novel pathway through deregulating cell metastasis and imply that HMGA2 might be used as a new prognostic marker and/or as an effective therapeutic target for HCC.

## Introduction

Hepatocellular carcinoma (HCC) is the seventh most general cancer in the world and the third leading origin of death among cancers worldwide. And, the highest incidence in the world has arisen in Asia and Africa [1]. In particular, hepatitis B virus (HBV) infection is the major factor in chronic liver disease and HCC. Therefore, HBV infection remains a global health issue with eventful morbidity and mortality [2–4] The HBV X protein (HBx) is an oncogenic protein that is required for infection and replication of the virus and is closely related to the development of HCC. Therefore, the HBx is considered to take a pivotal part in the molecular pathogenesis of HBV-correlative HCC [5–7]. HBx promotes migration and invasion of HCC cells through upregulating osteofibronectin and matrix metalloproteinases to reduce intercellular adhesion [8, 9]. Multiple reports have verified that HBx can operate numerous cancer-related genes, and pilot the formation and progression of tumors [10–13]. Hence, HBx has an essential position during HCC progression resulting in HBV chronic infection [14, 15]. However, the complex mechanisms of HBX regulating tumor development are still not well known.

Enhancer of zeste homolog 2 (EZH2) is a catalytic subunit of the Polycomb Repressor Complex 2 (PRC2), having the form of a complex with SUZ12 and EED protein. This complex catalyzes the trimethylation of histone H3 lysine 27 (H3K27me3), which restrains the transcription of many genes. The function of EZH2 participates in a heavy role in the regulation of genes during biological development, especially in cancer, where abnormal performance is often observed [16, 17]. During the past decade, EZH2 has been identified as an oncogene in many types of cancers and acts through epigenetic repression of various tumour suppressor genes [18–23]. Besides, inhibiting the transcription of many genes, EZH2 has also been presented to suppress the expression of many tumor suppressor microRNAs (miRNAs), such as miR-125a, miR-125b, miR-138-5p, and miR-320c in HCC, thus assisting HCC tumorigenicity and metastasis [24–27].

In this study, we wanted to understand the relationship between the molecular mechanism of between HBx and EZH2 in promoting HCC. In addition, we addressed the functional interplay between HBx and the miRNA machinery to identify potential initiating events for HCC metastasis and to identify the mechanism by which HBx and EZH2 regulate these genes.

## Results

### EZH2 is frequently overexpressed in HCC patients and correlated with overall survival

**Fig. 1:**
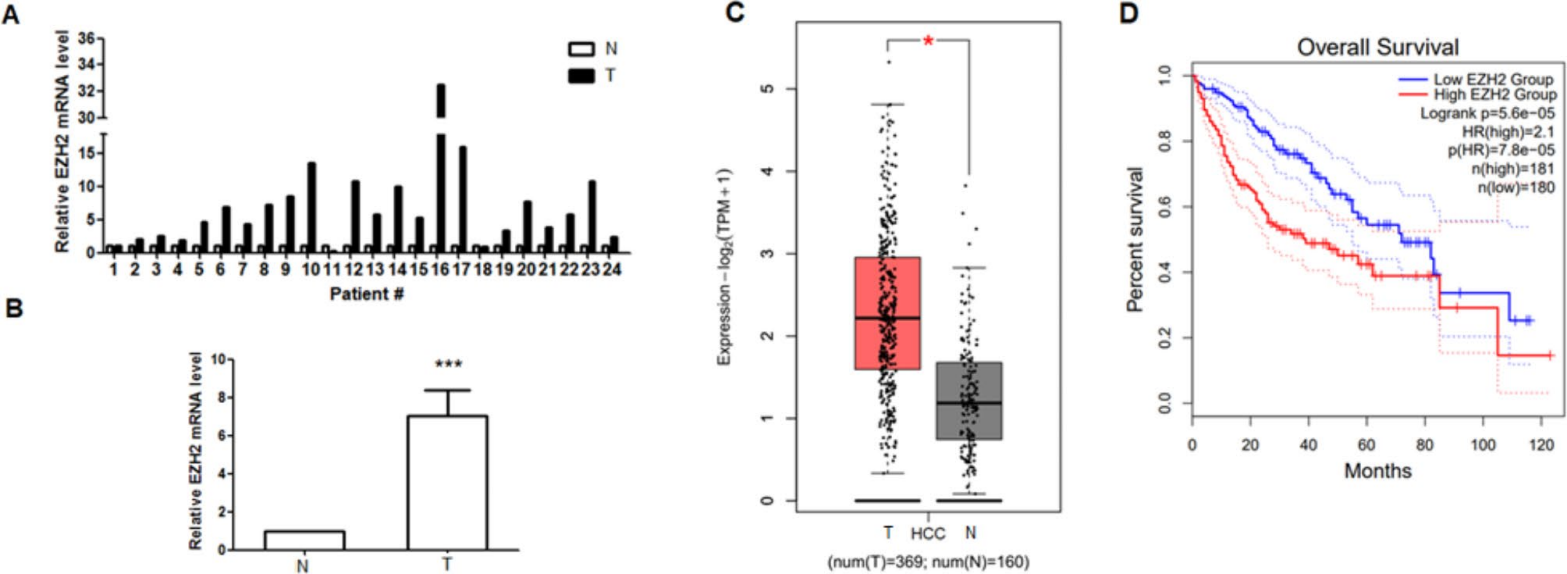
Analysis of EZH2 expression in HCC tumor samples. **A**. EZH2 mRNA relative to actin (control) was measured by qRT-PCR in 24 HCC tumor tissue samples in contrast to adjacent normal liver tissue from the same patients. **B**. The mean ± SEM of the relative EZH2 mRNA level for all 24 cases. **C**. The level of EZH2 between HCC tumors and normal control. **D**. The overall survival of EZH2 in HCC patients from TCGA database. (*p < 0.05, ***p < 0.001)

We verified the expression of EZH2 in 24 pairs of HBV-related HCC tumor tissues compared to normal adjacent liver tissue. This study assessed the levels of EZH2 mRNA through qPCR. The comparative levels of EZH2 in the tumor section and the normal tissues (n = 24) are shown in Fig. 1A. In most patients (21/24, 87.5%), EZH2 was upregulated in HCC tissues relative to normal adjacent tissues, with a mean ± SEM fold increase of 7.01 ± 1.39 (P < 0.001, Fig. 1B). The EZH2 levels in 20 of 24 tumor tissue samples (83.3%) were increased by ≥ 2-fold in contrast to in normal adjacent tissues. Futhermore, in order to investigate the clinical impact of EZH2 on HCC progression, we also assessed the relationship between cellular levels of EZH2 mRNA and the overall suvival in HCC. HCC patients with higher EZH2 expression compared to the normal control had significant results and high level of EZH2 indicated poor suvival (Fig. 1C, D). Based on the above data, we speculated that the overexpression of EZH2 may be related to HBV infection.

### HBx upregulates EZH2 expression

**Fig. 2:**
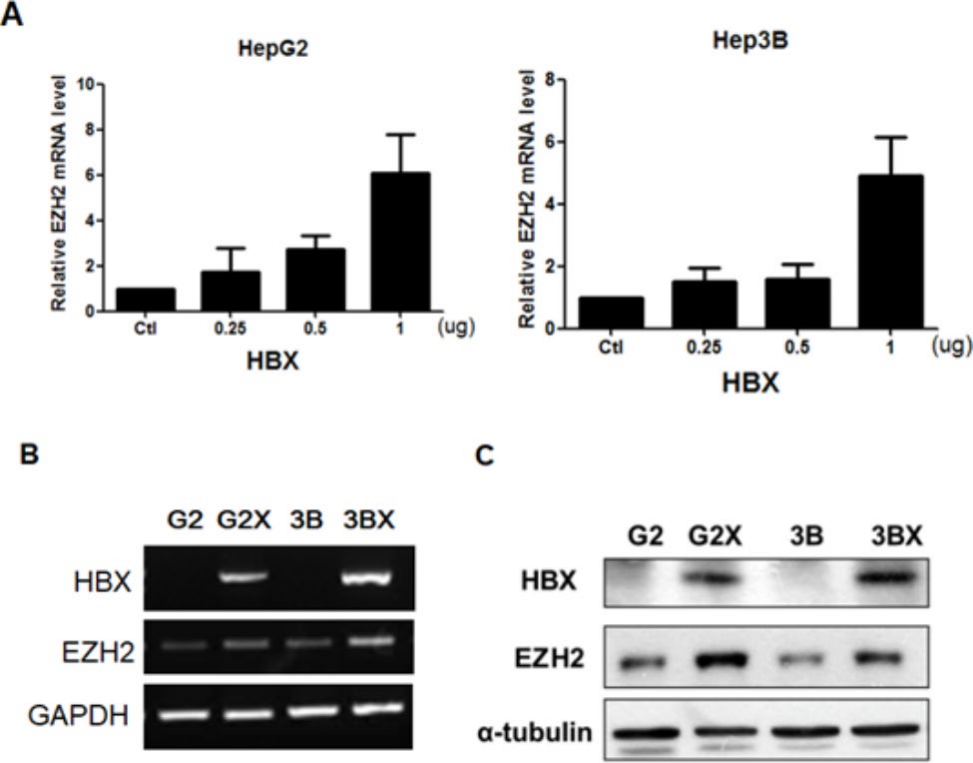
Upregulation of EZH2 proportion by HBx. **A**. HepG2 and Hep3B cells were temporarily transfected with a control vector plasmid (Ctl) or an HBx expression plasmid at the indicated concentration. EZH2 expression was surveyed by qRT-PCR. **B**. RT-PCR analysis of EZH2 expression in HepG2X (G2X) and Hep3BX (3BX) cells in contrast to parental cells HepG2 (G2) and Hep3B (3B), respectively. **C**. Western blot analysis of EZH2 protein in HepG2X and Hep3BX cells in contrast to parental cells HepG2 and Hep3B. The GAPDH and α-tubulin as controls

To determine whether the proportion of EZH2 is correlative to HBx overexpression, we used an encoding HBx plasmid into HepG2 and Hep3B cells on two human HCC cell lines. The expression of EZH2 was obviously elevated in HBx-expressing HepG2 and Hep3B cells as compared with the empty vector (Fig. 2A). In addition, we applied HepG2X and Hep3BX, which stably overexpress HBx[10, 11], to determine the proportion of HBx in contrast to their parental cell lines HepG2 and Hep3B, separately. EZH2 mRNA levels were heightened in HepG2X and Hep3BX cells as compared with the parental cells (Fig. 2B). Similarly, the level of EZH2 protein was also raised in HepG2X and Hep3BX cells (Fig. 2C). The results provided evidence that HBx upregulates EZH2 expression in HCC cell lines.

### let-7c and miR-99a are key regulatory miRNAs in HBV-related HCC

**Fig. 3:**
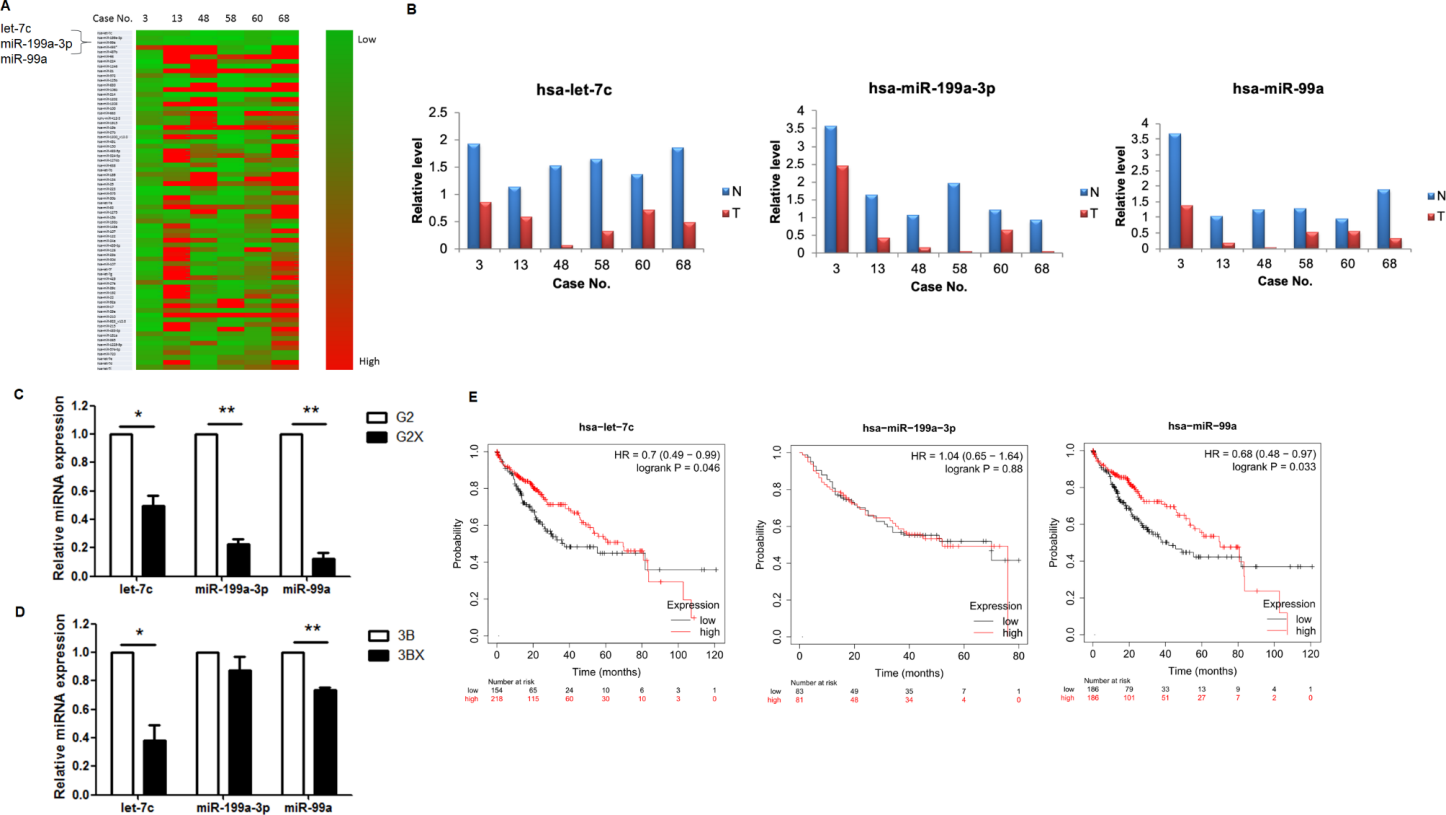
The profile of miRNAs downregulated by HBx. **A**. Heatmap of 72 miRNAs that were differentially expressed in six HBV-related HCC tumors (case number 3, 13, 48, 58, 60, 68) as compared with their non-tumor tissue controls. qRT-PCR analysis of these three miRNAs was performed **B** in the HCC tumor (T) and non-tumor (N) tissue samples and **C** in HepG2X and **D** in Hep3BX cells as compared with the parental cells HepG2 (G2) and Hep3B (3B), respectively. **E**. The overall survival of let-7c, mir-199a-3p, and mir-99a in HCC patients from TCGA database

To verify the influence of HBx on miRNA representation, we examined miRNA levels in HCC tissues gathered from six patients in contrast to HBV-related HCC tumors with non-tumor tissue samples. Seventy-two miRNAs were found to present remarkable changes in expression in all six paired biological samples (Fig. 3A). Only three miRNAs were downregulated in all six HCC tissue samples, let-7c, miR-99a, and miR-199a-3p (Fig. 3B). To identify whether these regulated miRNAs were thought of together with HBx in HCC cells, we verified their relative levels of expression in HepG2X and Hep3BX cells through qRT-PCR. let-7c and miR-99a levels were lower in the rates of HepG2X and Hep3BX cells relative to the parental cells (Fig. 3C, D). Besides, the level of miRNA-199a-3p was decreased only in HepG2X cells as compared with the parental cells. Simultaneously, a tricistronic miRNA cluster bearing miR-99a/let-7c/miR-125b-2 resides on hsa21. Moreover, to investigate the clinical impact of let-7 C, mir-199a-3p, and mir-99a on HCC progression. The overall suvival of these miRNAs had been evaluated with the KM plotter and the resulut indicated that lower let-7c and mir-99a have poor survial in HCC patients (Fig. 3E). These consequences supported an opinion that HBx downregulates the expression of let-7c and miR-99a.

### EZH2 represses let-7c and miR-99a expression

**Fig. 4:**
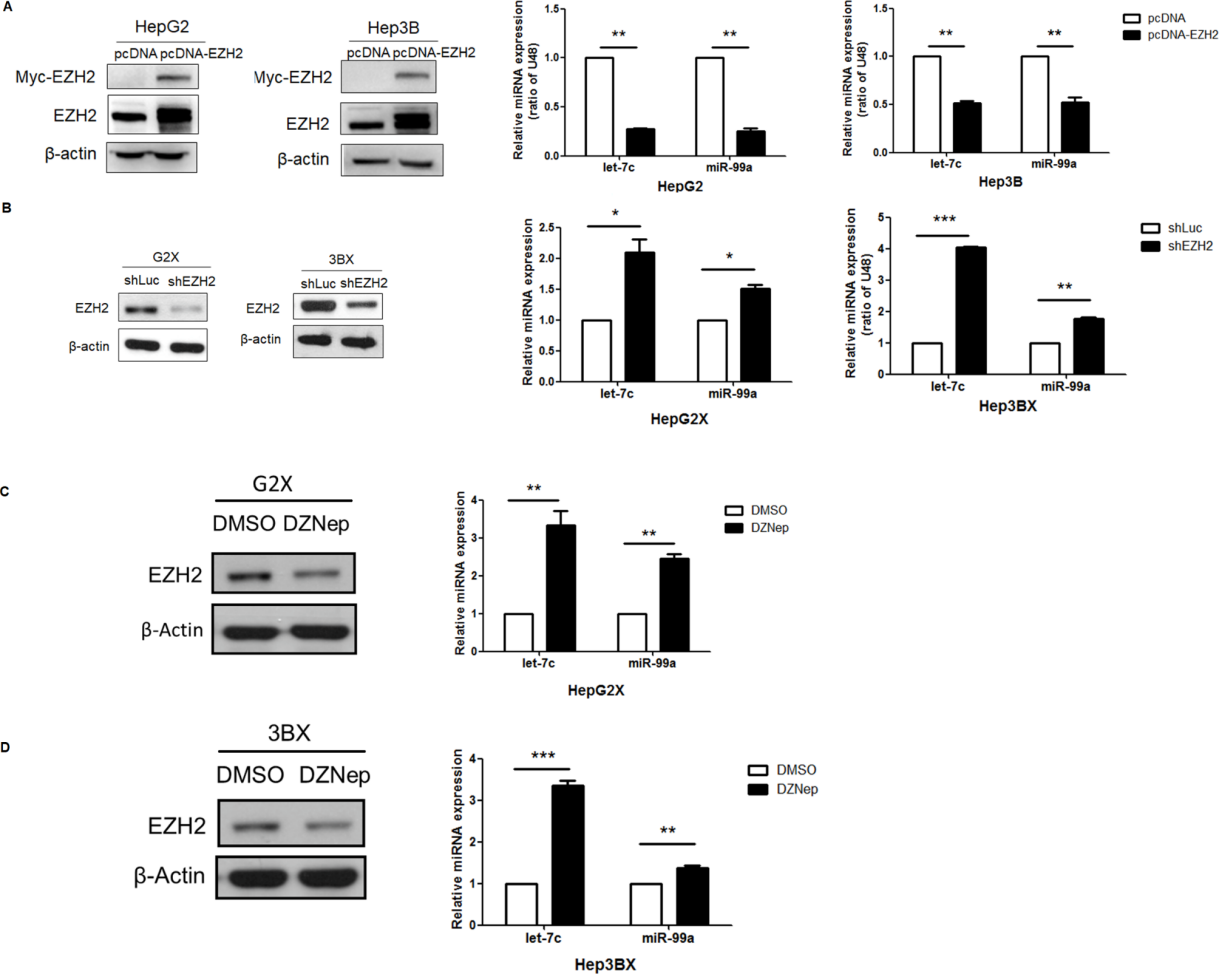
Analysis of EZH2 repression of let-7c and miR-99a expression. **A**. EZH2 was transfected into HepG2 and Hep3B cells. The expression of EZH2 was elevated in EZH2-expressing HepG2 and Hep3B cells as compared with cells transfected with the empty vector. let-7c and miR-99a expression was normalized against the control miRNA U48, as measured using qRT-PCR. **B**. The expression of let-7c and miR-99a was elevated in HepG2X and Hep3BX cells after knockdown of EZH2 by shRNAs. **C**. HepG2X and **D** Hep3BX cells handled with DZNep (10 µM) or DMSO (vehicle control) for 48 h. were executed to western blot analysis with anti-EZH2. The expression of let-7c and miR-99a was also determined in the same cells by qRT-PCR.

For the purpose of determining whether the expression of microRNAs is repressed because of EZH2 overexpression, we utilized a plasmid encoding EZH2 into HepG2 and Hep3B cells. The level of EZH2 was considerably elevated in EZH2-expressing HepG2 and Hep3B cells in contrast to the empty vector (Fig. 4A). To investigate the phenomenon of EZH2 knockdown, we executed short hairpin RNA (shRNA) experiments in HepG2X cell lines. For this purpose, we applied shRNAs that targeted positions in the EZH2 mRNA nucleotide sequence. Consistent with the results, let-7c and miR-99a expression were significantly increased in EZH2 knockdown HepG2X cells relative to the parental cells (Fig. 4B). let-7c evidenced a greater fold increase than miR-99a in EZH2 knockdown cells. An inhibitor of S-adenosylhomocysteine hydrolase, DZNep, leads to the maintenance of S-adenosylhomocysteine, accordingly restraining S-adenosyl-L-methionine dependent methyltransferases as well as EZH2 [28, 29]. DZNep also reportedly depletes EZH2 protein [30]. As expected, HCC cells handled with DZNep (10 µM) declared a substantial decline in EZH2 expression and showed a corresponding significant increase in let-7c and miR-99a representation (Fig. 4C, D). These results identified that DZNep enhances let-7c and miR-99a representation in HepG2X and Hep3BX cells.

### HBx-enhanced EZH2 expression and increases the binding in the let-7 promoter

**Fig. 5:**
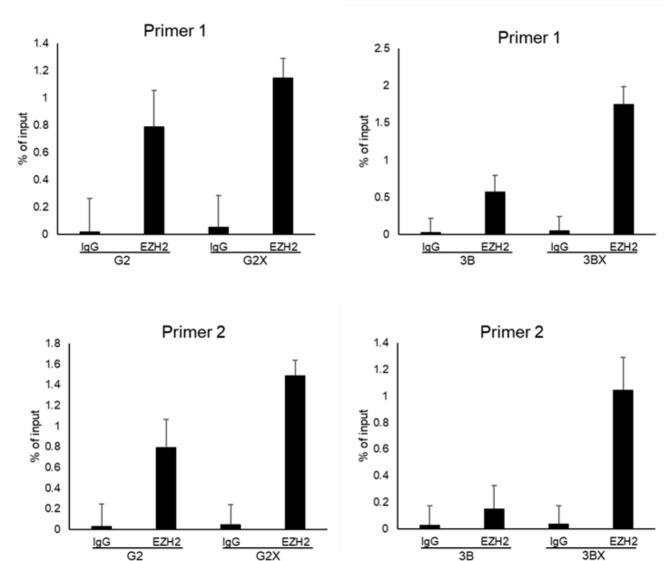
EZH2 binds to the let-7 promoter induced by HBx overexpression cell lines. ChIP assay using antibodies against EZH2 to detect binding to the let-7c promoter in HepG2, HepG2X, Hep3B, and Hep3BX cells

miRNA expression has an effect on epigenetics [31] as well as histone and DNA methylation are coadjutant associated with epigenetic regulatory mechanisms [32]. For the purpose of exploring the underlying mechanism of let-7c dysregulation by EZH2, we first identified whether EZH2 can bind the let-7c promoter. Moreover, to evaluate the efficacy of upregulated EZH2 on the let-7c promoter, a chromatin immunoprecipitation (ChIP) assay was executed. Two separate primer pairs were constructed that are both specific for the let-7c promoter. The ChIP consequences illustrated that EZH2 was directly relative to the let-7c promoter (Fig. 5). These findings declared that HBx-upregulated EZH2 may strengthen the binding in the let-7 promoter.

### HMGA2 is a target of let-7c

**Fig. 6:**
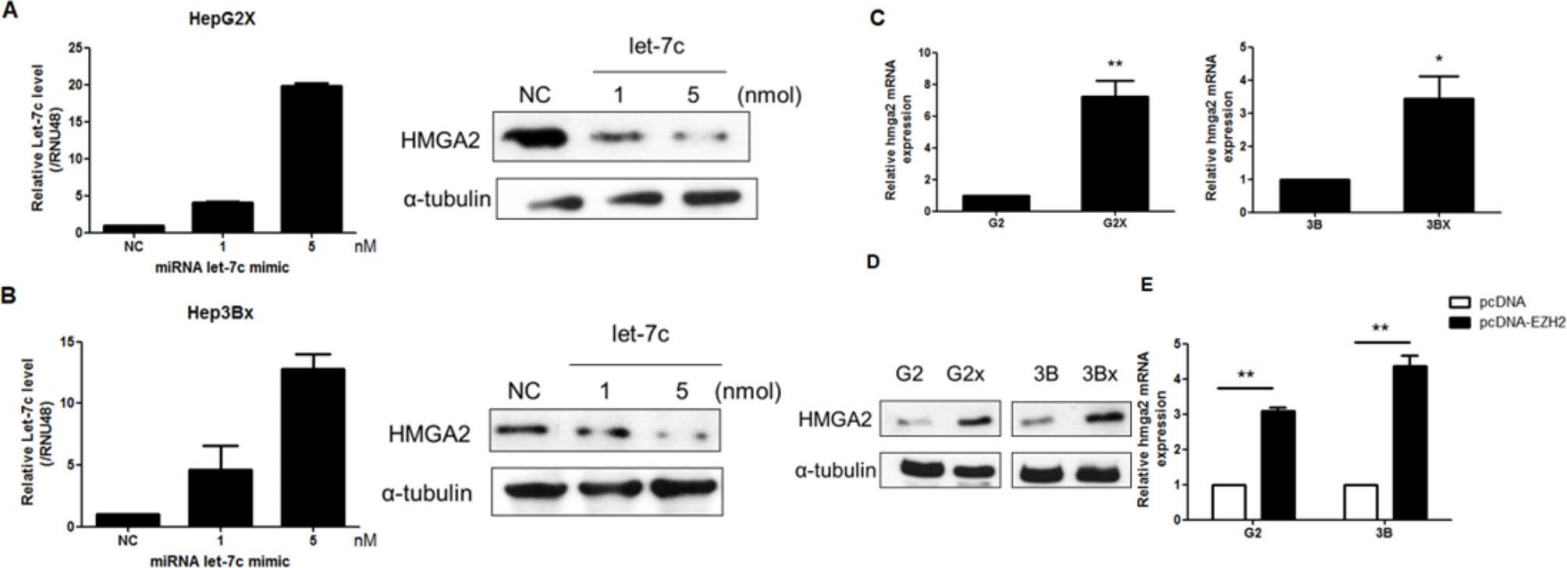
HMGA2 is a target of let-7c. **A, B**. The comparative HMGA2 protein levels in HepG2X and Hep3BX cells 48 h after transfection with the let-7c mimic or the miRNA mimic control (negative control, NC). α-Tubulin was adopted as the endogenous control. **C, D**. Differential expression of HMGA2 mRNA and protein was characterized in HepG2, HepG2X, Hep3B, and Hep3BX cells. **E** EZH2 was transfected into HepG2 and Hep3B cells. HMGA2 expression, which was measured using qRT-PCR, was normalized against β-actin

let-7c has been described to target HMGA2, a noticeable cellular oncogene [33]. Therefore, we investigated whether let-7c could downregulate HMGA2. We heightened let-7c expression through transfecting a let-7c mimic into HepG2X and Hep3BX cells while applying a mimic control as the negative control. A notable decline in the expression of HMGA2 protein was discovered after the upregulation of let-7c (Fig. 6A, B). Later, we examined whether the expression of HMGA2 corresponds to HBx expression in cells. HMGA2 mRNA levels were extraordinarily raised in HepG2X and Hep3BX cells in contrast to the parental cells (Fig. 6C). Likewise, HMGA2 protein levels were also significantly raised in HepG2X and Hep3BX cells (Fig. 6D). Furthermore, we adopted a plasmid encoding EZH2 into HepG2 and Hep3B cells. The result revealed that the level of HMGA2 was remarkably upraised in EZH2-expressing HepG2 and Hep3B cells in contrast to the empty vector (Fig. 6E).

### HBx promotes the migration of HCC cells via HMGA2

**Fig. 7:**
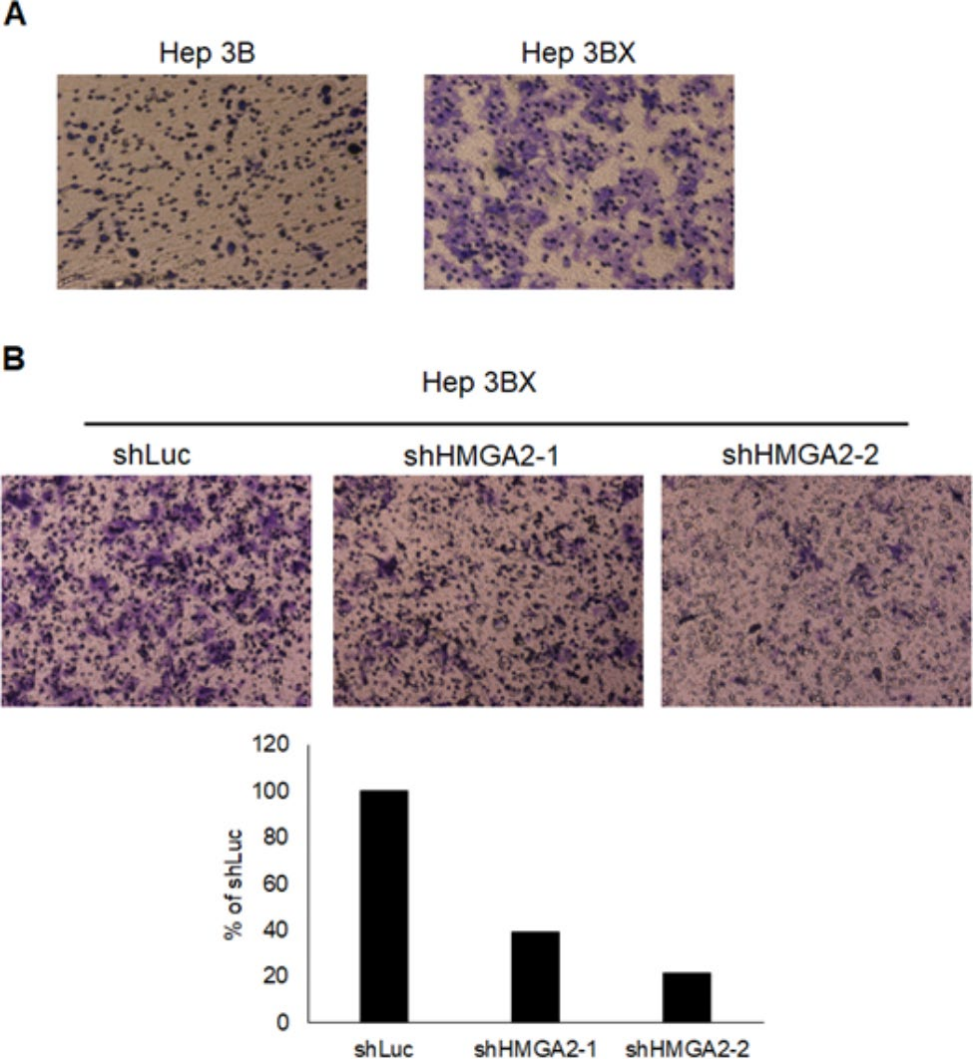
Verification of the capability of HBx on HCC cell migration. **A**. Migration ability was surveyed in Hep3B and Hep3BX cells with the Transwell migration assay. **B**. Hep3BX cells were examined with shLuc, shHMGA2-1, or shHMGA2-2, and their migration ability was then determined

To evaluate whether HBx mediates a heightening in the migration of HCC cells, we first compared the migration ability with Hep3B and Hep3BX cells. Our consequences confirmed that HBx accelerates cell migration (Fig. 7A). Moreover, the regulatory effect of HMGA2 on migratory capability was inspected with the transwell migration assay. Knockdown of HMGA2 with two individual shRNAs blocked the migration of Hep3BX cells (Fig. 7B). These data denoted that HMGA2 stands in the regulation of HCC cell migration.

## Discussion

In recent years, there has been significant progress and improvement in the research and treatment of hepatocellular carcinoma (HCC). Single-cell approaches are powerful tools for studying HCC at a cellular level. They enable the investigation of intracellular signaling pathways in individual tumor cells, providing insights into cellular heterogeneity and signaling network activation. Shi et al. (2012) developed a single-cell proteomic chip that allowed the profiling of signaling pathways in HCC cells. This technology identified cell-to-cell variations in pathway activation, revealing the heterogeneity of HCC. Advancements in single-cell technologies, such as mass cytometry and single-cell RNA sequencing (scRNA-seq), have further expanded our understanding of HCC signaling networks [34]. Mass cytometry enables the measurement of multiple protein markers in individual cells, facilitating the analysis of signaling pathway activation across different HCC cell types [35]. scRNA-seq has been extensively used to study HCC, allowing for the simultaneous profiling of gene expression in thousands of cells and providing insights into cell populations and communication networks. It has been demonstrated that the application of scRNA-seq in analyzing the tumor microenvironment and drug efficacy in HCC, uncovering specific cellular interactions and molecular signatures [36]. Therefore, single-cell approaches enable the study of cellular heterogeneity, identification of rare cell populations, and characterization of signaling protein activation at a single-cell resolution, ultimately advancing our understanding of HCC development, progression, and therapeutic responses. Hepatitis B x antigen (HBx) has an extensive aspect of functions for regulating many genes in the host, it participates in the signaling pathways such as cell proliferation, cell cycle, metastasis, invasion, and protein degradation [37]. In our previous research, HBx was reported to be associated with HCC cell migration [10]. Therefore, HBx is a critical risk factor and a therapeutic target for the development of HCC [38]. In this study, we observed a significantly higher mRNA expression of EZH2 in most tumor samples in a contrast to non-tumor tissues. Similarly, Sasaki and Sudo also declared that EZH2 is highly expressed in cancer tissues and is closely related to the degree of tumor malignancy [39, 40]. Additionally, Xiao-Yan Shi et al. found that HBx raises the expression of EZH2 in hepatocytes through activation of the transcription factor E2F1 [41]. There are results that HBx can induce liver fibrosis also through the inhibition of the EZH2 complex [25]. There are results that HBx can induce liver fibrosis also through the inhibition of the EZH2 complex. And also, EZH2 complex acts as a pivotal regulator of epigenetics in HCC and promotes tumor growth and metastasis during HCC development [42, 43].

miRNA dysregulation can be discovered in many classes of cancer, and involved in the invasion and metastasis of cancer. Hence, miRNAs may act as tumor suppressors or oncogenes [44]. It is known that EZH2 as an epigenetic regulator, is involved in solid tumors progression and metastasis. EZH2 was reported to inhibit let-7c, miR-101, miR-125b, miR-139, and miR-200b [25, 45, 46]. In this study, we searched out the regulatory mechanism of EZH2 and discovered that HBx can depress the expression of cellular miRNAs (let-7c, miR-199a, and miR-99a) to adjust these critical cellular pathways. Henry et al. reported a remarkable reduction in miR-199a-3p expression and targeted by rapamycin (mTOR) and c-Met in HCC cell lines [47]. Hence, miR-199a-3p was shown to own tumor suppressor function. In addition, miR-99a has commonly been restrained in various tumors such as tongue squamous cell carcinoma, lung cancer, bladder, and prostate cancer [48–51]. And, the restrained capability of miR-99a on the development of HCC has been illustrated in vitro and in vivo [47]. Furthermore, IGF-1R and mTOR are first-hand objectives of miR-99a, indicating the property of miR-99a as a cell cycle inhibitor [52]. let-7, the first miRNA discovered in humans, involves in many cancer-related genes such as c-MYC, HMGA2, and CCND1 [33]. The functions of let-7 are well known to participate in the development, proliferation, and differentiation. According to our consequences, we found that HBx and EZH2 were highly expressed in HBV-infected HCC, and EZH2 adjusts methylation of the let-7 promoter, thereby suppressing the level of let-7.

**Fig. 8:**
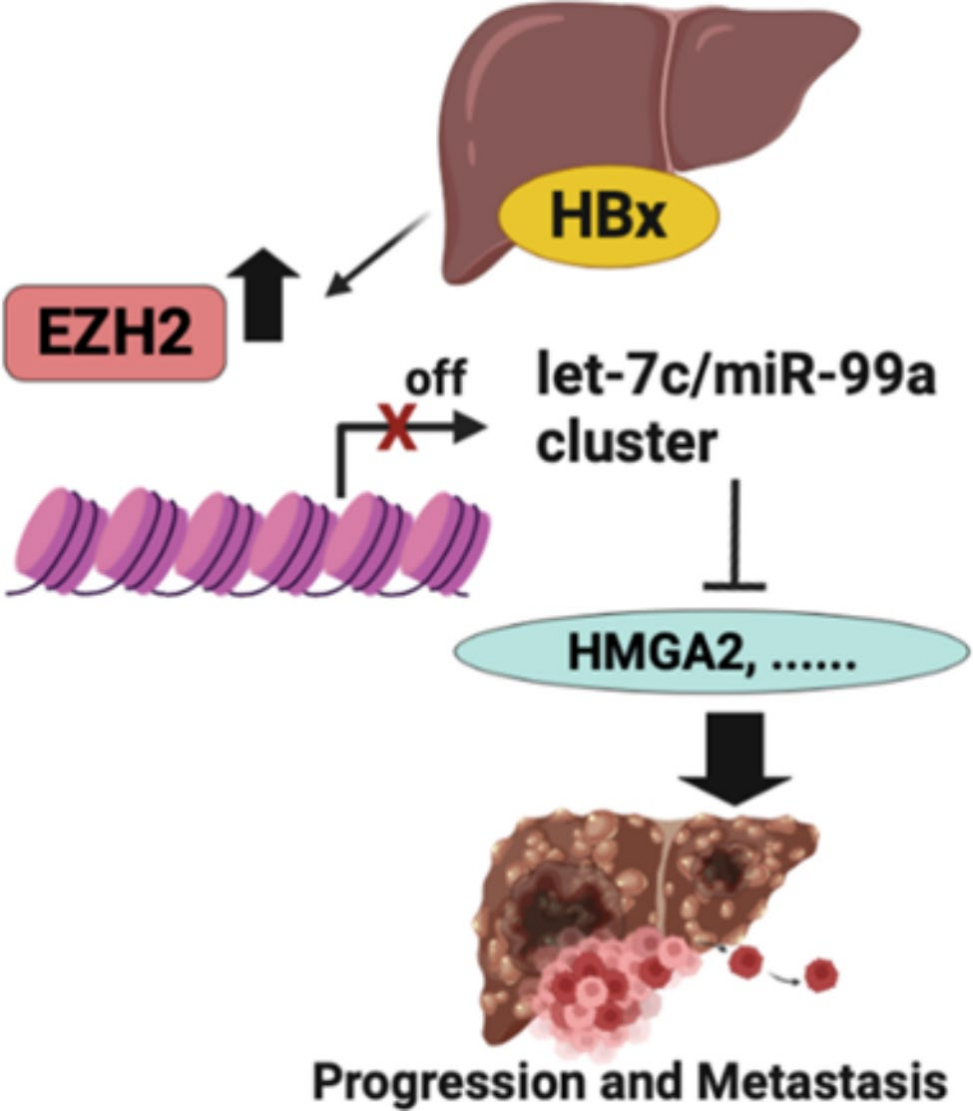
A proposed illustration for the molecular mechanism of HBx-induced HCC migration. The newly identified HBx/EZH2/let-7c/HMGA2 pathway suggests a molecular mechanism that leads to HCC cell migration

HMGA2 binds to AT-rich regions in DNA and converts chromatin structure. It also interacts with certain proteins, such as E2 promoter-binding factor (E2F1) combing to create a complex to rise gene transcription [53]. During early developmental periods, HMGA2 is widely presented in many undifferentiated cells. AS fetal development goes on, the level of HMGA2 reduces [54–56]. Nevertheless, in most malignant tumors, the expression of HMGA2 raises, such as breast, lung, and pancreas cancer [57–59]. Importantly, the increased expression of HMGA2 is relative to accelerating angiogenesis, strengthening EMT, invasion, and metastasis in cancer progression [53, 60]. HMGA2 can effectively adjust EMT through the PI3K/AKT, MAPK/ERK, TGFB/Smad, NFrB, and STAT3 signaling pathways, enabling HCC cells to invade local tissues, get the penetrating ability of intravascular, and give birth to offspring with the tumor-initiating ability [53]. Therefore, in our results, the HMGA2 mRNA levels were obviously raised in HCC cells (HepG2X and Hep3BX) in contrast to the parental cells through the EZH2 - let-7c axis. We also discovered that the knockdown of HMGA2 restrained the migrated capability of Hep3BX. Besides, we confirmed that the down-regulation of let-7c causes the elevation of HMGA2 and improves the metastasis of HCC (Fig. 8). And, we provided new insights into the EZH2 epigenetic repression of let-7c that advances HCC migration caused by HBx. In summary, our research supported two markers, let-7c and HMGA2, as diagnostic markers and probable therapeutic objectives for HBV-infected HCC.

## Conclusions

Our results provide new insights into the EZH2 epigenetic repression of let-7c that promotes HCC migration induced by HBx. Thus, let-7c and HMGA2 represent key diagnostic markers and potential therapeutic targets for the treatment of HBV-related HCC.

## Methods

### Patient characteristics

Serum samples were received from the National Cheng Kung University Hospital in Taiwan. Sample identified information was encrypted to guard patient confidentiality. And the samples obeyed the agreements approved by the Institutional Review Board of Cheng Kung University Hospital (assignment number: ER-99-176).

### Comprehensive analysis of EZH2, let-7c, mir-199a-3p, and mir-99a from the Cancer Genome Atlas (TCGA)

Gene expression profile interactive analysis (GEPIA, http://gepia.cancer-pku.cn/index.html) uses standard processing pipelines to analyze RNA-Seq expression data from GTEx and TCGA. The Kaplan-Meier plotter can assess the survival rate of the effect of 54k genes (mRNA, miRNA, protein) and 21 cancer types (http://kmplot.com/analysis/). Sources for the databases include GEO, EGA, and TCGA. In this study, we used GEPIA for tumor/normal differential expression and overall survival analysis of EZH2 and the KM plotter for overall survival analysis of let-7c, mir-199a-3p, and mir-99a in HCC.

### Microarray analysis

The mirVana miRNA Bioarray V9.2 was acquired from Ambion. The experimental method is according to the manufacturer’s protocol. Briefly, miRNAs were elicited with the mirVana™ miRNA Isolation Kit and were condensed with a flashPAGE™ Fractionator. After, small RNAs containing mature miRNAs were tagged with the mirVana™ miRNA Labeling Kit and hybridized to the mirVana™ miRNA Bioarray V9.2.

### Cell Culture

HepG2 and Hep3B cell lines were gained from the American Type Culture Collection (Maryland), cultured in Dulbecco’s modified Eagle’s medium (DMEM)/F12 medium supplemented with 10% fetal bovine serum (HyClone, Logan, UT). HepG2X and Hep3BX cells are apart derivatives of human hepatoma HepG2 and Hep3B cells, and stably express the gene encoding HBx, as reported previously [61].

### Plasmids and miRNA mimic

The pcDNA-EZH2 plasmid was purched from Addgene (Cambridge, MA). The let-7c mimic and the miRNA mimic control were acquired from Dharmacon (Lafayette, CO). The miRNA mimic is a double-stranded oligonucleotide devised to mimic the function of an endogenous mature miRNA.

### Transient transfection

Transfections were executed with Lipofectamine 2000, based on the manufacturer’s manual. After 48 h., cells were applied for subsequent related experiment [62].

### Gene knockdown with shRNAs

Knockdown of genes was executed with specific shRNAs conveyed with the lentiviral system from the National RNAi Core Facility (Academia Sinica, Taipei, Taiwan) on the basis of their instruction manual. The shRNA constructs targeting HMGA2 are clones TRCN0000021966 and TRCN0000342671, and the construct targeting EZH2 is clone TRC0000040076. The shRNA construct against luciferase (shLuc), clone TRCN0000072244, was adopted as a negative control. The capability and the specificity were examined according to the previous report [11].

### Quantitative PCR (qPCR)

Total RNA was extracted with TRIzol reagent (Invitrogen) based on the manufacturer’s instructions. The primers (Table 1) were synthesized by Invitrogen. qPCR was executed as previously described [11, 63], and detected with the LightCycler 480 apparatus (Roche).

**Table 1:** The primer sequence of genes for PCR and QPCR

Gene	Sequence	
PCR		
HBx forward	GTTAAGCTTATGGCTGCTAGGCTGTGCTGC	
HBx reverse	AGACTCGAGCCGGGCAGAGGTGAAAAAGTTGC	
EZH2 forward	GCAGTAAAAATGTGTCCTGCAAGAA	
EZH2 reverse	TCAAGGGATTTCCATTTCTCTTTCGA	
GAPDH forward	CATCCCTGCCTCTACTGGCG	
GAPDH reverse	AGGCCATGTGGGCCATGAGG	
**QPCR**		**UPL probe**
HBX forward	TTGGGGGAGGAGATTAGGTT	75
HBX reverse	TGGTGAACAGACCAATTTATGC	
EZH2 forward	GACTGGCGAAGAGCTGTTTT	64
EZH2 reverse	TCTTTCGATGCCGACATACTT	
HMGA2 forward	TCCCTCTAAAGCAGCTCAAAA	34
HMGA2 reverse	ACTTGTTGTGGCCATTTCCT	
β-actin forward	ATTGGCAATGAGCGGTTC	11
β-actin reverse	GGATGCCACAGGACTCCAT	
has-let-7c-RT	GTTGGCTCTGGTGCAGGGTCCGAGGTATTCGCACCAGAGCCAACAACCAT	
has-let-7c forward	GGCCATGAGGTAGTAGGTTGT	21
has-miR-99a-RT	GTTGGCTCTGGTGCAGGGTCCGAGGTATTCGCACCAGAGCCAACCACAAG	
has-miR-99a forward	GGCAAAACCCGTAGATCCGAT	21
has-miR-199a-3p-RT	GTTGGCTCTGGTGCAGGGTCCGAGGTATTCGCACCAGAGCCAACTAACCA	
has-miR-199a-3p forward	GGCACACAGTAGTCTGCACAT	21
U48-RT	GTTGGCTCTGGTGCAGGGTCCGAGGTATTCGCACCAGAGCCAACTCAGCG	
has-U48 forward	CGGCGGTAACTCTGAGTGTGT	21
miRNA reverse	GTGCAGGGTCCGAGGT	

### Chromatin immunoprecipitation (ChIP) assay

A ChIP assay was executed by the MAGnify Chromatin Immunoprecipitation kit (Invitrogen). The experimental procedure was obeyed by the manufacturer’s manual. Finally, the break of chromatin was immunoprecipitated with EZH2 antibodies overnight at 4 °C. Two sets of primers were designed on the report of the let-7c promoter, and PCR acted as described [62]. The two primer pairs were as follows: primer pair 1, AGCCTTCTGAGCCAGTTTCTTC (forward) and CCCGAGCAGTAGCAGTGTG (reverse); primer pair 2, CGGGTGCTTTCTATCTCTTCTCC (forward) and TGGATGCCGTGGCTTCTCG (reverse).

### Western blot analysis

The antibodies and chemicals were applied: anti-hepatitis B Virus X antigen (Abcam, Burlingame, CA, USA; 1:1000); anti-EZH2, clone AC22 (Cell Signaling; 1:1000); anti-HMGA2 (Abcam; 1:1000); and anti-β-actin (Sigma; 1:5000); Chemiluminescence detection reagent (GE, Piscataway, NJ). The experimental procedure refers to the previous report [11].

#### Transwell Migration assays

Cell migration was evaluated with Boyden chambers (Millipore, Billerica, MA). In brief, 1 × 10^5^ cells in DMEM plus 1% FBS were seeded onto each membrane insert (8 μm). After 24 h incubation, the cells in the upper chamber were dismissed with a cotton swab. The invasive cells were then stained with Giemsa and calculated under a light microscope at 100x magnification [64, 65].

### Statistical analysis

Detailed examination of data was calculated with Prism 8 (GraphPad) and Excel (Microsoft). Statistical analyses were carried out with a two-tailed Student’s t-test or Fisher’s exact test.

## Data Availability

Not applicable.
